# Editorial: Insight into long working hours: perspective from the construction of intelligent society

**DOI:** 10.3389/fpsyg.2023.1241070

**Published:** 2023-08-18

**Authors:** Qingqing Sun, Hong Chen, Bei Liu, Yanki Hartijasti

**Affiliations:** ^1^School of Economics and Management, China University of Mining and Technology, Xuzhou, Jiangsu, China; ^2^School of Business, Jiangnan University, Wuxi, Jiangsu, China; ^3^Research Institute of National Security and Green Development, Jiangnan University, Wuxi, China; ^4^University of Indonesia, Depok, Indonesia

**Keywords:** long working hour, intelligent society, time poverty, mental health, psychological mechanism

## Introduction

In the past few decasdes, topics such as “overwork” and “time poverty” have attracted a lot of attention (Wu et al., 2019). According to the Global Working Environment Survey published by the International Labor Organization, long working hours (more than 48 h per week) and short breaks (< 11 h between working days) are common worldwide (Khed and Krishna, 2023). “Working long hours” has become a “social disease” that needs to be addressed (Spector et al., 2017). In particular, as the construction of a smart society continues, digital, networked and intelligent working conditions have led to a deepening and integration of the connections between social agents (Zhao and Yu, 2023). This, moreover, poses a new set of challenges in guiding employees' motivation and behavior at work.

This Research Topic aims to explore the psychological mechanisms behind the long working hours of employees driven by new technologies, new industries and new models, with an emphasis on interdisciplinary contributions, in the context of building a smart society. This Research Topic currently contains five manuscripts on “how after-hours work connectivity affects employees' sense of exuberance at work and at home,” “an evaluation of psychosocial risk factors for teleworking,” “the impact of the ‘odd job economy' on the mental health of migrant workers,” “the impact of overtime on mental health,” “the relationship between changes in work/sleep patterns and mental distress”. Specific innovative findings are as follows.

## Specific innovative findings

Yang et al. extended the scenario boundaries of work connectivity behavior after-hours (WCBA) from a passive adaptation perspective. This study not only complements the positive effect of WCBA on work exuberance, but also reveals the double-edged effect of WCBA on work exuberance. Yang et al. suggest that in adopting WCBA, organizations need to be mindful of the sensitivity of employees' different family needs to interconnected work, and that targeted measures must be taken to enhance work-family enrichment and mitigate work-family conflict.

Since the start of the COVID-19 pandemic thousands of people have experienced teleworking and this practice is becoming increasingly commonplace. Antunes et al. used “telecommuting” and “frequency” (“part-time” or “full-time”) and their synonyms and variants as keywords and their synonyms and variants were used as keywords for the database search and analysis. The results found significant differences in psychosocial risk factors of concern to scholars before and after the COVID-19 epidemic. Most of the studies conducted prior to the COVID-19 pandemic focused more on the intensity of working hours, social relationships at work, etc. Research conducted during the COVID-19 pandemic focused more on the emotional needs of workers for their families while telecommuting. The manuscript also suggests that it is crucial to define limits on the duration and intensity of work. Full-time telecommuting brings important changes to working conditions that have the potential to affect not only the lives and health status of remote workers, but also to positively influence psychosocial risk factors and facilitate work-family balance and communication.

Xu et al. focused on vulnerable groups of migrant workers in China. Empirically examined the relationship between on-call work and depression among migrant workers, providing a basis for psychological crisis intervention for migrant workers in China. She recommends that in order to alleviate and prevent depression among migrant workers, targeted mental health management plans should be developed, depression assessment should be incorporated into routine health screening, and mental health-related services should be provided to migrant workers with high depression indices. Yao explored the mediating role of social support and work value consciousness between overtime work and physical and mental health of Chinese workers.

Tondokoro et al. confirmed that regardless of working hours, shortened sleep time may be a key factor in psychological distress. This study highlights the importance of sleep management in maintaining employees' mental health and the need to consider the circumstances and conditions of other daily tasks, such as working hours, in order to better manage sleep.

## Perspectives

In summary, the relevant papers included in this Research Topic not only provide relevant investigations on the psychological mechanism of long-term labor on employees in the context of a smart society, but also focus on the impact of working hours and work intensity on mental health, which helps to alleviate the impact of unlimited overflow of work boundaries on employees' health damage. It also provides new enlightenment on the impact of laborers and working hours on social and economic development, especially on the healthy development of a smart society from a macro and long-term perspective.

## Author contributions

All authors listed have made a substantial, direct, and intellectual contribution to the work and approved it for publication.
